# An Appeal for Prophylaxis of Venereal Diseases

**Published:** 1905-08

**Authors:** Louis C. Roughlin

**Affiliations:** Atlanta, Ga.


### ATLANTA
### Journal-Record of Medicine.

**Successor to Atlanta Medical and Surgical Journal, Established 1855, and Southern Medical Record, Established 1870.**

OWNED BY THE ATLANTA MEDICAL JOURNAL CO.

**Published Monthly.**

VOL. VII. AUGUST, 1905. No. 5.

BERNARD WOLFF, M.D., EDITOR, Nos. 319-20 Prudential.

M. B. HUTCHINS, M.D., BUSINESS MANAGER, 1007-1008 Century Bldg.

J. N. LeCONTE, M.D., ASSOCIATE EDITOR.

### ORIGINAL COMMUNICATIONS.

### AN APPEAL FOR PROPHALAXIS OF VENEREAL DISEASES.*

INDEX THROUGH

By LOUIS C. ROUGLIN, M.D., Atlanta, Ga.

Prior to the discovery of Neiser in 1879, that gonorrhea is produced by a specific germ, the gonococcus, an attack of the disease was considered a trivial matter both by the physician and laity, and the cessation of the discharge was considered the termination of the disease by patient and physician, and to-day when the physician has learned to recognize in the gonococcus as the most virulent, destructive and baneful organism which infects the human economy, the physician has so far failed to impress upon the mind of the laity and his patients the severity of the disease, the destructive consequence of its complications, the murderous and death-producing results of its sequellae, and we can yet but too often hear the unfortunate remark made by so many of the public, that "I'd rather have a case of clap than a cold."

That the cessation of the discharge is still considered as a cure

* Read before the Atlanta Medical Society June 1, 1905.

for the disease not only by the patient, but unfortunately encouraged in that belief to a large extent by the physician, is but too evident from the number of cases of latent gonorrhea and infected prostates that every physician sees in a too great number of cases previously discharged as "cured" by a physician, and the enormous sales of the advertised "sure cures" and by the "three-day cures" so much lauded by the public and often bragged about by the physician himself. That such erroneous ideas still exist and that such a prevalent scourge to the community, that such a murderous, death-dealing disease is allowed to progress, and to mutilate and debase its victims—not only the guilty, but the innocent, the child as well as the adult, the chaste and pure as well as the most corrupt and immoral, unbridled and unhindered by the hand of science or legislation—I regret, but I say it most conscientiously, that I feel I have no apologies to make, neither no exceptions to offer, when I say before you, a society of men of medicine and learning, you, and you alone, by your inactivity are responsible for that condition.

I know of no disease whose effects, remote and immediate, leads to more disastrous results than gonorrhea. In one of my examinations one of the questions was: "Name the complications and seqellae of gonorrhea." I began with the first letter of the alphabet, "A," and ended with "Z." I could have named more but for the lack of time. Dr. Ely Van de Warker, naming the triology of the diseases that urgently press for solution, in order of their importance, puts gonorrhea first and tuberculosis and syphilis later, and adds: "I place gonorrhea before tuberculosis not because it is less destructive to life than the latter, but because it is potentially more dangerous to society in its power to impair fertility and permanently infringe upon its usefulness." The disease claims for its victims not only the guilty, but its most serious results are often inflicted upon the innocent and virtuous. A woman by developing the disease threatens the prosperity and well being of the community.

Our text-books all agree that gonorrhea is the chief source of salpingitis, pelvic peritonitis and is responsible for the majority of gynecological cases. Bland Sutton, of London, states that gonorrhea is the chief source of sterility in the female, and I believe it is often the source of sterility in the male. Morrow, in his most valuable book on "Social Diseases and Marriage," states that "no disease has such a murderous influence on the offspring as syphilis, yet no disease has such a destructive influence on the health and procreative function of woman as gonorrhea." It is a disease as formidable as tuberculosis, and as much a social plague as syphilis. It possesses not only the dangerous traits of causing destructive processes by itself, but it has the power to develop non-pathogenic germs to malignant activity, and if we should measure this dangerous activity by its devastating effects, by its lasting physical and functional disabilities, we can not but see in this universal, ever-spreading scourge on humanity a serious menace and danger threatening the fertility and prosperity of the nation. Its effects are so destructive to the human organism that I fear not that any contradiction will be made when I shall express my conscientious belief that no disease known to medical science has caused so much indirect mortality, mutilation and suffering as gonorrhea, and the medical profession could hardly spend its time and valuable influence in a more humanitarian direction than in educating the public in regards to its severity and ravages, and suggesting means of limiting or preventing its progress in the future.

I fully realize that in dealing with prophylaxis of venereal diseases we have the most complex, the most baffling and difficult subject of a medico-sociological nature which confronts us at the present time. Much has been done and tried in that direction, yet nothing valuable has been accomplished.

Should we, then, in the light of previous failures and with the enormous task confronting us, remain inactive and indifferent? Should such be the attitude of the medical profession? Then, I am certainly gravely mistaken in the profound esteem of the humanitarian and courageous men which I hold to be true of the medical profession. Let us suggest such plans with which the community, every home, can be protected; and if but one suggestion should be made to-night in the discussion of this paper, which can be carried out and prove of practical utility, the aim of the speaker will be accomplished.

Let us but view what has been done in the past, so that we may be better able to judge what may be practical in the future. The following have been tried and found ineffective:

1. Enforced examination of prostitute.
2. Segregation of prostitute to certain quarters.
3. Commitment of prostitute to the lock hospital when diseased.
4. Licensing prostitution.

I shall not discuss the ineffectiveness and positive failure of the first three mentioned. It has proven itself unpractical and unbeneficial in the continent where it has been most rigidly enforced. It necessarily must be a failure, as all of the impositions have been put upon the women, and no restriction on the men who have the disease and are the common carriers of same and introduce it to the purest of homes. As to legalizing and licensing prostitution, a suggestion which we so often hear, I can not express my opposition to it nor my reason for same in any stronger terms than the following of Howard A. Kelly, who in speaking of the subject says:

"If we legalize this infamous business, where shall we look to recruit the ever-fading ranks of these poor creatures as they die yearly by the tens of thousands? Which of the little girls of the land shall we designate for this traffic? Mark their sweet innocence to-day as they run about our streets and parks, prattling and playing, ever busy about nothing, and earth's only memento of the angels in their guilelessness. Which of them shall we snatch as they approach maturity to supply the foul mart of the insatiable cravings of lust? Perish the thought! Again, we surely would not allow the daughters of our rich men to enter our legalized brothels. The poor man must suffer and be robbed of the flower of his family—the poor man who, Jacob Riis tells us, has not appeal beyond the policeman on the beat and practically no rights in our courts. What can he do against the lavish use of money by the rich man's son and the brothel keeper's oath and policeman? Sherman said, 'War is hell,' but war is a sweet, a noble and choice calling, compared with a life in this pit of iniquity which some have proposed to open in our land."

What then must we do? Permit me, gentlemen, to make a suggestion. First of all, we must educate the public so that it may become enlightened to the severity and dangers of the disease; for, to have a success in any form of venereal prophylaxis, we must obtain the full and sincere co-operation of the public at large—the clergyman and the lawyer, the merchant and mechanic, the common laborer as well as the physician; we must secure the co-operation of the public press, where articles suitable to be read by the general public should appear from time to time warning of the threatened danger to the community and nation. Lectures free to the public should be given by suitable persons on the subject, and the public be urged to attend. Let the children in school, as soon as they are deemed old enough, be taught the danger which confronts them; let us impress firmly in their minds that the "Wages of sin is death," and in this instance, a living death worse than hell. Meanwhile, let us put literature in the hands of our patients urging the use of antiseptics after every suspicious intercourse. In short, let us put the public, beginning with the child, and continuing with the adult, where they may realize the true position of this ever-spreading, devastating calamity, and the battle will be won.

The task is before you, gentlemen! It is your duty! Will you perform it? It concerns you personally, your sons, your homes. Let, in the discussion, be brought forward suggestions which can be practically carried out; and above all, let not the discussion end it. Let us do something.

809–811 English-American Building.

---

### Sprains.

Dr. Brinton recommends that the limb is to be put into a vessel of very hot water immediately, boiling water being added as it can be borne, and kept immersed for twenty minutes, or until the pain ceases. Then put on a pretty tight bandage and order rest. Sometimes the joint can be used in twelve hours. If the trouble is more chronic, apply a silicate of sodium dressing, and let the patient walk with a cane, if the ankle be the joint affected.

